# Asymptomatic Giant Right Renal Oncocytoma: A Case Report

**DOI:** 10.7759/cureus.34129

**Published:** 2023-01-24

**Authors:** Gökhan Çevik, Atınç Tozsin, Ezgi G Erdoğan, Hakan Çakıcı, Hakan Akdere

**Affiliations:** 1 Urology, Sultan 1. Murat State Hospital, Edirne, TUR; 2 Urology, T.C. (Türkiye Cumhuriyeti) Trakya Üniversitesi, Trakya Üniversitesi Hastanesi, Edirne, TUR; 3 Pathology, T.C. (Türkiye Cumhuriyeti) Trakya Üniversitesi, Trakya Üniversitesi Hastanesi, Edirne, TUR; 4 Urology, Optimed Hospital, Tekirdağ, TUR

**Keywords:** radical nephrectomy, giant oncocytoma, open nephrectomy, renal cell carcinoma (rcc), renal oncocytoma

## Abstract

Renal oncocytoma is usually detected incidentally. It can be considered as a renal cell carcinoma (RCC) on preoperative imaging. They usually present as small masses and usually look like benign tumors. Giant oncocytomas are rare. A 72-year-old male patient was seen in the outpatient department for left scrotal swelling. Ultrasound (US) showed a giant mass compatible with RCC in the right kidney which was incidentally detected. Abdominal computed tomography (CT) revealed a mass with an axial diameter of 167×146 mm, compatible with RCC, a heterogeneous mass of soft tissue density with central necrosis. There was no evidence of tumor thrombus in the right renal vein or inferior vena cava. Open radical nephrectomy was performed through an anterior subcostal incision. Pathological examination revealed a 17×15 cm renal oncocytoma. The patient was discharged on the sixth day postoperatively. Clinically or radiologically, renal oncocytoma and renal cell carcinoma usually cannot be distinguished, although oncocytoma may be suspected if a central scar with fibrous extensions is seen, the so-called "spoke-wheel appearance". The treatment decision should be made according to the clinical aspects. Radical/partial nephrectomy or thermal ablation can be considered as treatment options. In this article, we review the literature on the radiological and pathological features of renal oncocytoma.

## Introduction

Renal oncocytomas are frequently smaller than renal cell carcinomas and generally present asymptomatically. However, these lesions usually cannot be distinguished, although oncocytoma may be suspected if a central scar with fibrous extensions is seen, the so-called "spoke-wheel appearance". The similarity of radiographic features between these lesions makes their clinical differentiation difficult. Recent series show that 3% to 7% of solid renal cortical tumors are oncocytomas [1]. Oncocytomas arise from the distal renal tubules, especially from the interrelated cells of the collecting tubules [2]. This case report emphasizes that oncocytomas should be included in the differential diagnosis of large renal masses. Oncocytomas usually present as small masses [3]. Large oncocytomas are rare, but the prognosis of giant oncocytomas has not been reported to be different from small lesions. In the literature, incidentally detected giant renal oncocytomas have been reported in a small number of patients [4,5].

## Case presentation

**Figure 1:**
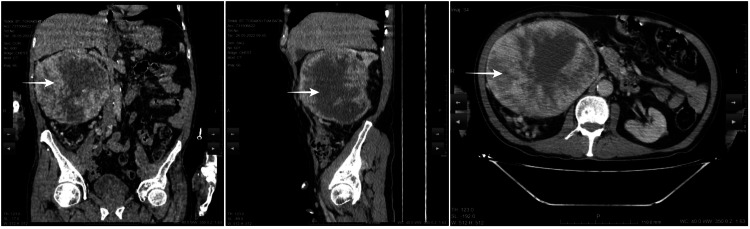
Contrast-enhanced computed tomography images Arrow indicates mass in the antero-mid-lower part of the right kidney with an axial diameter of 167×146 mm, a heterogeneous mass of soft tissue density with central necrosis, consistent with RCC.

A 72-year-old male patient was seen in the outpatient department because of left scrotal swelling. He did not complain of any pain. Physical examination revealed no pathology other than left scrotal swelling. Urinary system ultrasound (US) was performed and revealed a giant mass compatible with RCC in the right kidney. A hydrocele was detected in the left scrotum. Routine laboratory tests including renal functions were in normal values. Contrast-enhanced abdominal computed tomography (CT) revealed a mass in the antero-mid-lower part of the right kidney with an axial diameter of 167×146 mm, a heterogeneous mass of soft tissue density with central necrosis, consistent with RCC (Figure 1).

**Figure 2:**
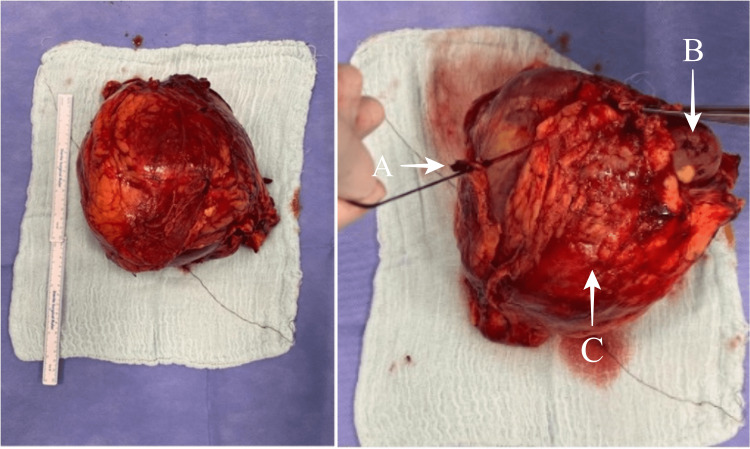
Macroscopic pictures of mass A: Ureter; B: normal kidney parenchyma; and C: mass.

Dilated vessels were observed adjacent to the mass. Due to findings compatible with RCC, an open radical nephrectomy was performed through the right anterior subcostal incision. There were no postoperative complications. The patient was discharged on the sixth day postoperatively. On macroscopic examination of the nephrectomy material, the right kidney measured 19×18×9.5 cm and had a 6.3 cm ureter above it (Figure 2).

**Figure 3:**
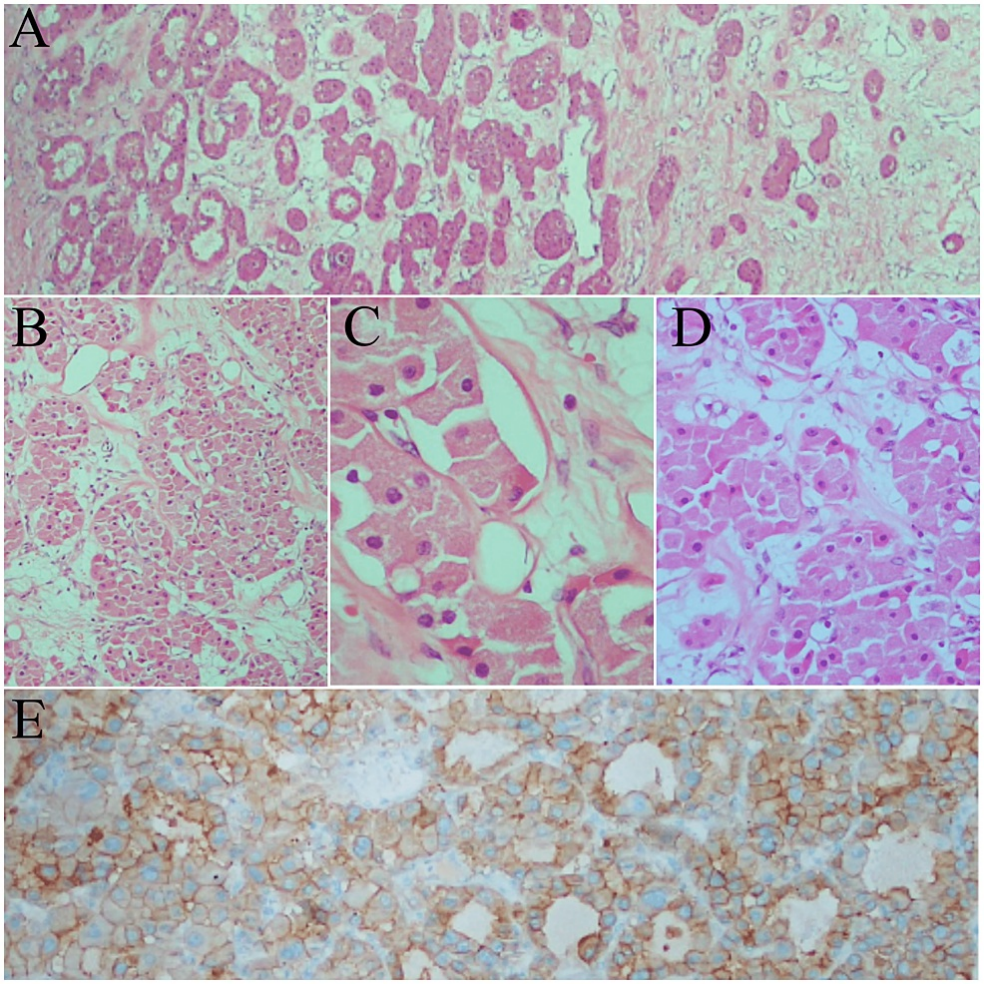
Histopathological examination of the tumor A. Solid-nested architecture and small islands of oncocytoma ×40 hematoxylin and eosin (H&E). B. Solid-nested architecture of oncocytoma ×100 H&E. C. The eosinophilic cells with granular cytoplasm and round nuclei ×400 H&E. D. The large eosinophilic cells of the oncocytoma ×200. E. The tumor shows immunoreactivity for CD117 (KIT) ×100 (immunohistochemistry).

A dirty yellow-gray colored tumoral formation with necrosis and bleeding foci of 15×17 cm, adjacent to the renal sinus and pelvis, was observed, pushing the renal parenchyma aside. Macroscopically, the tumor did not invade the perinephric adipose tissue. Microscopic examination revealed a well-circumscribed tumor consisting of small solid islands with myxoid stroma. The tumor cells were large, round, eosinophilic cells with dense granular cytoplasm. Immunohistochemistry showed that CD117 was positive; vimentin, CD10, and CAIX19 were negative; and scattered rare positive cells were detected with CK7. The final diagnosis was a stage II (pT2bNxMx) oncocytic renal neoplasm with low malignant potential (Figures 3A-3E).

## Discussion

After it was first described by Zippel in 1942, Klein and Valensi published a case series covering the benign course and different pathological features of oncocytoma [6]. Renal oncocytoma originating from distal tubule cells, also the source of renal cell carcinoma, is the second most common solid tumor in the kidney. It has similar histological features to the eosinophilic variant of chromophobe carcinoma. It is frequently not possible to differentiate by clinical and radiological criteria. Usually, these lesions are found incidentally on investigation for other reasons. Although a central star-shaped scar and spoke-wheel pattern on the supplying arteries are common imaging findings, they are unreliable for differential diagnosis. The most typical finding is the spoke-wheel or stellate pattern detected in the arterial phase of renal angiography [7,8]. However, neither of these two findings is specific and diagnostic, and both findings can be seen during the evaluation of RCC. Treatment of these tumors may vary according to the clinical conditions. It can be treated with radical/partial nephrectomy or thermal ablation. Even large oncocytomas are rarely invasive; usually, the capsular margin is preserved. In cases of rapid growth and concomitant RCC, oncocytomas should be closely monitored and treated [9]. Macroscopically, they are cortically located, light brown-colored, homogeneous tumors. As the tumor grows, it has a central scar as a result of the avascular area. It consists of oncocytes originating from cortical intercalated cells. It may show cellular atypia or pleomorphism (12%-30%). Other atypical histological features can include bleeding (20%-30%) and perinephric fat invasion (11%-20%) [10]. The incidence of giant oncocytomas, which can be termed as 15 cm and above on the short axis, is not known exactly [11,12]. Recently, these tumors have been diagnosed when they are small and asymptomatic due to the advances in radiological imaging methods and their frequent use. The largest oncocytoma in the literature measured 27×20×15 cm and weighed 4,652 g [13]. Banks et al. reported the second-largest renal oncocytoma (3,090 g, 21×18×15 cm) [12]. Large renal oncocytomas can be asymptomatic. The largest treated asymptomatic renal oncocytoma (3,353 g, 20 cm) was reported by Sundararajan et al. [5]. Qaid et al. reported treating one renal oncocytoma (3,500 g, 15×16×195 cm) [4]. In our case, the tumor dimensions were 15×17 cm and the weight was 2,380 g.

The role of CT-guided biopsy for smaller renal lesions has been investigated, but the value of biopsy for larger lesions is limited. However, in small renal masses, the surveillance or treatment protocol can be discussed as a result of whether the material taken in the preoperative percutaneous core biopsy is benign or malign. Thus, the complicated consequences of radical surgery in benign lesions can be avoided [14]. In the current case, the preoperative percutaneous core biopsy was not considered because the mass was very large and radical treatment was planned (Table 1). Considering the inability to differentiate with preoperative imaging methods, it seems that the current literature recommends aggressive treatment for these tumors [15].

**Table 1:** Largest reported renal oncocytomas

Reference	Age	Sex	Tumor Size (cm)	Presenting Complaint	Treatment
Levine and Huntrakoon [13]	64	Male	27×20×15	Palpable abdominal mass	Right open radical nephrectomy
Ahmad et al. [11]	61	Male	25×16×16	Lower back/flank pain	Right open radical nephrectomy
Banks et al. [12]	57	Male	21×18×15	Abdominal discomfort and distension	Right open radical nephrectomy
Kilic et al. [16]	65	Male	20×25×10	Abdominal pain	Left open radical nephrectomy
Sundararajan et al. [5]	37	Male	20	Abdominal mass and moderate hypertension	Right open radical nephrectomy
Qaid et al. [4]	40	Male	15×16×19.5	Abdominal pain	Left open radical nephrectomy
Dey et al. [17]	59	Female	14×13×12	Palpable mass	Right open radical nephrectomy
Current case	72	Male	15×17	Left scrotal swelling	Right open radical nephrectomy

## Conclusions

Given the benign nature of renal oncocytomas, these interventions may be seen as overtreatment. However, these therapeutic approaches are justified as a definitive diagnosis is often not available preoperatively. However, the nature of the mass can be determined by preoperative percutaneous core biopsy if it will affect the follow-up or treatment decision. Oncocytomas behave as benign tumors, and the prognosis after current treatment modalities is excellent. No case of recurrence at the resection site has yet been published. In conclusion, it is difficult to differentiate renal oncocytoma from RCC on preoperative radiology images, especially when a large mass is present. The definitive diagnosis can only be made by histopathological examination of the mass.
